# Piperazine-1,4-diium bis­(perchlorate) dihydrate

**DOI:** 10.1107/S1600536810030345

**Published:** 2010-08-04

**Authors:** Cong-hu Peng

**Affiliations:** a Department of Chemical and Environmental Engineering, Anyang Institute of Technology, Anyang 455000, People’s Republic of China

## Abstract

The asymmetric unit of the title compound, C_4_H_12_N_2_^2+^·2ClO_4_^−^·2H_2_O, contains half of a piperazinediium cation, one perchlorate anion and one water mol­ecule. The diprotonated piperazine ring, which is completed by crystallographic inversion symmetry, adopts a chair conformation. In the crystal structure, the cations and anions are linked by inter­molecular N—H⋯O and O—H⋯O hydrogen bonds into a three-dimensional network.

## Related literature

For background to simple mol­ecular–ionic crystals containing organic cations and acid radicals (1:1 molar ratio), see: Katrusiak & Szafrański (1999 ▶, 2006 ▶).
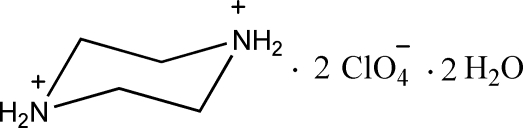


## Experimental

### 

#### Crystal data

C_4_H_12_N_2_^2+^·2ClO_4_^−^·2H_2_O
*M* *_r_* = 323.09
Monoclinic, 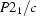

*a* = 7.2588 (15) Å
*b* = 6.5089 (13) Å
*c* = 14.543 (4) Å
β = 113.56 (3)°
*V* = 629.8 (3) Å^3^
*Z* = 2
Mo *K*α radiation
μ = 0.56 mm^−1^
*T* = 293 K
0.28 × 0.26 × 0.20 mm

#### Data collection

Rigaku Mercury 2 diffractometer
Absorption correction: multi-scan (*CrystalClear*; Rigaku/MSC, 2005 ▶) *T*_min_ = 0.856, *T*_max_ = 0.896
6362 measured reflections
1458 independent reflections
1130 reflections with *I* > 2σ(*I*)
*R*_int_ = 0.060

#### Refinement

*R*[*F* ^2^ > 2σ(*F* ^2^)] = 0.045
*wR*(*F* ^2^) = 0.109
*S* = 1.07
1458 reflections
83 parameters
H-atom parameters constrained
Δρ_max_ = 0.28 e Å^−3^
Δρ_min_ = −0.25 e Å^−3^

### 

Data collection: *CrystalClear* (Rigaku/MSC, 2005 ▶); cell refinement: *CrystalClear*; data reduction: *CrystalClear*; program(s) used to solve structure: *SHELXS97* (Sheldrick, 2008 ▶); program(s) used to refine structure: *SHELXL97* (Sheldrick, 2008 ▶); molecular graphics: *SHELXTL* (Sheldrick, 2008 ▶); software used to prepare material for publication: *SHELXL97*.

## Supplementary Material

Crystal structure: contains datablocks I, global. DOI: 10.1107/S1600536810030345/pv2304sup1.cif

Structure factors: contains datablocks I. DOI: 10.1107/S1600536810030345/pv2304Isup2.hkl

Additional supplementary materials:  crystallographic information; 3D view; checkCIF report

## Figures and Tables

**Table 1:** Hydrogen-bond geometry (Å, °)

*D*—H⋯*A*	*D*—H	H⋯*A*	*D*⋯*A*	*D*—H⋯*A*
N1—H1*B*⋯O5^i^	0.90	2.00	2.875 (3)	165
N1—H1*A*⋯O5^ii^	0.90	2.14	2.883 (3)	140
N1—H1*A*⋯O3^iii^	0.90	2.49	3.060 (3)	122
N1—H1*A*⋯O2^iv^	0.90	2.56	3.040 (3)	114
O5—H5*WB*⋯O3^v^	0.77	2.26	2.999 (3)	161
O5—H5*WA*⋯O1	0.82	2.59	3.192 (3)	131
O5—H5*WA*⋯O4	0.82	2.26	3.040 (3)	159

Symmetry codes: (i) 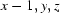; (ii) 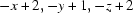; (iii) 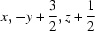; (iv) 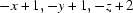; (v) 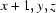.

**Fig. 1.**
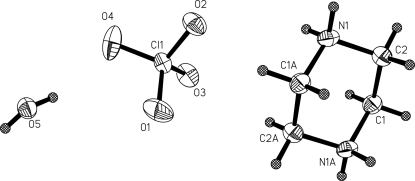
The asymmetric unit of the title compound with atomic labels. Displacement ellipsoids were drawn at the 30% probability level.

**Fig. 2.**
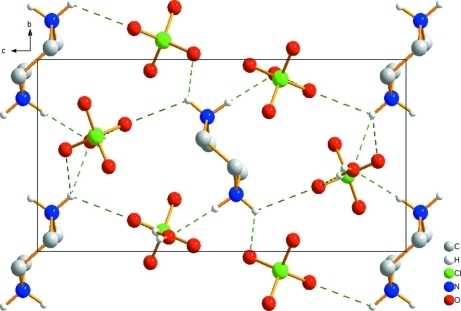
The unit cell packing of the title compound viewed along the a-axis; hydrogen bonds are drawn as dashed lines.

## supplementary crystallographic information

### Comment

Recently, much attention has been devoted to simple molecular–ionic crystals containing organic cations and acid radicals (1:1 molar ratio) due to the tunability of their special structural features and their interesting physical properties (Katrusiak & Szafrański, 1999; Katrusiak & Szafrański, 2006). In our laboratory, the title compound has been synthesized and its crystal structure is reported herein.

The asymmetric unit of the title compound consists of a half piperazine cation, one chlorate anion and one water molecule (Fig. 1). The diprotonated piperazine ring adopts a chair conformation. In the crystal structure, cations and anions are linked by intermolecular N—H···O and O—H···O hydrogen bonds into a three-dimensional network (Tab. 1 & Fig. 2).

### Experimental

Piperazine (1.7 g, 20 mmol) and 10% aqueous HClO_4_ (25 ml) in a molar ratio of 1:1 were mixed and dissolved in 30 ml water by heating to 353 K forming a clear solution. The reaction mixture was cooled slowly to room temperature, block crystals of the title compound were formed after fifteen days.

### Refinement

The H atoms of piperzinium ion were placed in calculated positions, with C—H = 0.97 and N—H = 0.90 Å, and refined using a riding model, with *U*_iso_(H) = 1.2*U*_eq_(C/N). The hydrogen atoms of the water molecule were located from a difference fourier map and were fixed at those positions with *U*_iso_(H)=1.5*U*_eq_(O).

### Crystal data

**Table:**

C_4_H_12_N_2_^2+^·2ClO_4_^−^·2H_2_O	*F*(000) = 336
*M**_r_* = 323.09	*D*_x_ = 1.704 Mg m^−^^3^
Monoclinic, *P*2_1_/*c*	Mo *K*α radiation, λ = 0.71073 Å
Hall symbol: -P 2ybc	Cell parameters from 1130 reflections
*a* = 7.2588 (15) Å	θ = 3.1–27.5°
*b* = 6.5089 (13) Å	µ = 0.56 mm^−^^1^
*c* = 14.543 (4) Å	*T* = 293 K
β = 113.56 (3)°	Block, colorless
*V* = 629.8 (3) Å^3^	0.28 × 0.26 × 0.20 mm
*Z* = 2	

### Data collection

**Table:**

Rigaku Mercury 2 diffractometer	1458 independent reflections
Radiation source: fine-focus sealed tube	1130 reflections with *I* > 2σ(*I*)
graphite	*R*_int_ = 0.060
Detector resolution: 13.6612 pixels mm^-1^	θ_max_ = 27.5°, θ_min_ = 3.1°
ω scans	*h* = −9→9
Absorption correction: multi-scan (*CrystalClear*; Rigaku/MSC, 2005)	*k* = −8→8
*T*_min_ = 0.856, *T*_max_ = 0.896	*l* = −18→18
6362 measured reflections	

### Refinement

**Table:**

Refinement on *F*^2^	Secondary atom site location: difference Fourier map
Least-squares matrix: full	Hydrogen site location: inferred from neighbouring sites
*R*[*F*^2^ > 2σ(*F*^2^)] = 0.045	H-atom parameters constrained
*wR*(*F*^2^) = 0.109	*w* = 1/[σ^2^(*F*_o_^2^) + (0.0357*P*)^2^ + 0.361*P*] where *P* = (*F*_o_^2^ + 2*F*_c_^2^)/3
*S* = 1.07	(Δ/σ)_max_ < 0.001
1458 reflections	Δρ_max_ = 0.28 e Å^−^^3^
83 parameters	Δρ_min_ = −0.25 e Å^−^^3^
0 restraints	Extinction correction: *SHELXL97* (Sheldrick, 2008), Fc^*^=kFc[1+0.001xFc^2^λ^3^/sin(2θ)]^-1/4^
Primary atom site location: structure-invariant direct methods	Extinction coefficient: 0.044 (4)

### Special details

**Table:**

Geometry. All e.s.d.'s (except the e.s.d. in the dihedral angle between two l.s. planes) are estimated using the full covariance matrix. The cell e.s.d.'s are taken into account individually in the estimation of e.s.d.'s in distances, angles and torsion angles; correlations between e.s.d.'s in cell parameters are only used when they are defined by crystal symmetry. An approximate (isotropic) treatment of cell e.s.d.'s is used for estimating e.s.d.'s involving l.s. planes.
Refinement. Refinement of *F*^2^ against ALL reflections. The weighted *R*-factor *wR* and goodness of fit *S* are based on *F*^2^, conventional *R*-factors *R* are based on *F*, with *F* set to zero for negative *F*^2^. The threshold expression of *F*^2^ > σ(*F*^2^) is used only for calculating *R*-factors(gt) *etc*. and is not relevant to the choice of reflections for refinement. *R*-factors based on *F*^2^ are statistically about twice as large as those based on *F*, and *R*- factors based on ALL data will be even larger.

### Fractional atomic coordinates and isotropic or equivalent isotropic displacement parameters (Å^2^)

**Table:**

	*x*	*y*	*z*	*U*_iso_*/*U*_eq_	
C1	0.3021 (4)	1.0866 (4)	0.94987 (19)	0.0411 (6)	
H1C	0.2516	1.0117	0.8870	0.049*	
H1D	0.1989	1.1821	0.9491	0.049*	
C2	0.3493 (4)	0.9393 (4)	1.03562 (19)	0.0426 (6)	
H2B	0.3904	1.0149	1.0981	0.051*	
H2A	0.2303	0.8603	1.0272	0.051*	
Cl1	0.78737 (8)	0.59992 (10)	0.84112 (4)	0.0390 (2)	
N1	0.5133 (3)	0.7985 (3)	1.03919 (15)	0.0401 (5)	
H1B	0.4715	0.7234	0.9825	0.048*	
H1A	0.5420	0.7115	1.0912	0.048*	
O1	0.9198 (3)	0.7709 (4)	0.87194 (18)	0.0765 (7)	
O2	0.7472 (3)	0.5298 (3)	0.92385 (14)	0.0561 (6)	
O3	0.6016 (3)	0.6622 (3)	0.76257 (13)	0.0562 (6)	
O4	0.8781 (4)	0.4421 (4)	0.80648 (18)	0.0853 (8)	
O5	1.3089 (2)	0.5745 (3)	0.85594 (12)	0.0421 (5)	
H5WB	1.3600	0.6040	0.8207	0.063*	
H5WA	1.1857	0.5653	0.8324	0.063*	

### Atomic displacement parameters (Å^2^)

**Table:**

	*U*^11^	*U*^22^	*U*^33^	*U*^12^	*U*^13^	*U*^23^
C1	0.0416 (14)	0.0393 (14)	0.0403 (14)	0.0025 (11)	0.0140 (11)	0.0007 (11)
C2	0.0444 (14)	0.0446 (15)	0.0407 (14)	−0.0009 (12)	0.0189 (12)	0.0031 (11)
Cl1	0.0336 (3)	0.0498 (4)	0.0351 (3)	−0.0020 (3)	0.0152 (2)	−0.0022 (3)
N1	0.0538 (13)	0.0292 (10)	0.0348 (11)	−0.0025 (9)	0.0149 (9)	0.0020 (9)
O1	0.0532 (13)	0.0832 (17)	0.0868 (16)	−0.0303 (12)	0.0214 (12)	0.0037 (13)
O2	0.0542 (11)	0.0726 (14)	0.0442 (11)	−0.0003 (10)	0.0226 (9)	0.0140 (10)
O3	0.0415 (10)	0.0814 (15)	0.0391 (10)	0.0024 (10)	0.0091 (9)	0.0105 (10)
O4	0.0803 (16)	0.102 (2)	0.0775 (17)	0.0328 (15)	0.0362 (14)	−0.0225 (14)
O5	0.0373 (9)	0.0469 (11)	0.0433 (10)	0.0015 (8)	0.0174 (8)	0.0026 (8)

### Geometric parameters (Å, °)

**Table:**

C1—N1^i^	1.487 (3)	Cl1—O1	1.421 (2)
C1—C2	1.500 (3)	Cl1—O2	1.4218 (19)
C1—H1C	0.97	Cl1—O3	1.4339 (19)
C1—H1D	0.97	N1—C1^i^	1.487 (3)
C2—N1	1.487 (3)	N1—H1B	0.90
C2—H2B	0.97	N1—H1A	0.90
C2—H2A	0.97	O5—H5WB	0.77
Cl1—O4	1.417 (2)	O5—H5WA	0.82
			
N1^i^—C1—C2	109.6 (2)	O4—Cl1—O2	110.46 (15)
N1^i^—C1—H1C	109.7	O1—Cl1—O2	109.05 (14)
C2—C1—H1C	109.7	O4—Cl1—O3	110.15 (14)
N1^i^—C1—H1D	109.7	O1—Cl1—O3	109.38 (14)
C2—C1—H1D	109.7	O2—Cl1—O3	108.62 (12)
H1C—C1—H1D	108.2	C2—N1—C1^i^	111.62 (19)
N1—C2—C1	109.7 (2)	C2—N1—H1B	109.3
N1—C2—H2B	109.7	C1^i^—N1—H1B	109.3
C1—C2—H2B	109.7	C2—N1—H1A	109.3
N1—C2—H2A	109.7	C1^i^—N1—H1A	109.3
C1—C2—H2A	109.7	H1B—N1—H1A	108.0
H2B—C2—H2A	108.2	H5WB—O5—H5WA	118.5
O4—Cl1—O1	109.16 (16)		

Symmetry codes: (i) −*x*+1, −*y*+2, −*z*+2.

### Hydrogen-bond geometry (Å, °)

**Table:**

*D*—H···*A*	*D*—H	H···*A*	*D*···*A*	*D*—H···*A*
N1—H1B···O5^ii^	0.90	2.00	2.875 (3)	165
N1—H1A···O5^iii^	0.90	2.14	2.883 (3)	140
N1—H1A···O3^iv^	0.90	2.49	3.060 (3)	122
N1—H1A···O2^v^	0.90	2.56	3.040 (3)	114
O5—H5WB···O3^vi^	0.77	2.26	2.999 (3)	161
O5—H5WA···O1	0.82	2.59	3.192 (3)	131
O5—H5WA···O4	0.82	2.26	3.040 (3)	159

Symmetry codes: (ii) *x*−1, *y*, *z*; (iii) −*x*+2, −*y*+1, −*z*+2; (iv) *x*, −*y*+3/2, *z*+1/2; (v) −*x*+1, −*y*+1, −*z*+2; (vi) *x*+1, *y*, *z*.
